# Immunological Predictors of Post Infectious Inflammatory Response Syndrome in HIV-Negative Immunocompetent Cryptococcal Meningitis

**DOI:** 10.3389/fimmu.2022.895456

**Published:** 2022-05-24

**Authors:** Yijie Wang, Hang Wei, Liping Shen, Xiaohong Su, Jia Liu, Xiaofeng Xu, Min Li, Lu Yang, Junyu Liu, Anni Wang, Ying Jiang, Fuhua Peng

**Affiliations:** ^1^ Department of Neurology, The Third Affiliated Hospital of Sun Yat-sen University, Guangzhou, China; ^2^ School of Medical Information Engineering, Guangzhou University of Chinese Medicine, Guangzhou, China

**Keywords:** cryptococcal meningitis, HIV-negative, immunocompetent, postinfectious inflammatory response syndrome, predictor

## Abstract

**Objective:**

This research aims to study the correlation between serum immune factors and post-infectious inflammatory response syndrome (PIIRS) in immunocompetent cryptococcal meningitis (CM), and explore whether serum immune factors could be used to predict the development of PIIRS.

**Methods:**

A cohort of 30 patients with PIIRS and 87 patients without PIIRS was selected from 347 CM patients. We analyzed the general clinical information and immunological indexes (cytokines, complement, immunoglobulin, inflammation, related cytological and biochemical indexes). Spearman correlation analysis and principal component analysis were used to explore the effects of the variables on PIIRS. Additionally, the variables were identified by a random forest-based classifier for predicting the development of PIIRS. The clinical value of predictors was verified by survival analysis.

**Results:**

Compared with patients without PIIRS, patients with PIIRS had lower baseline serum interleukin-6 (IL-6, P = 0.006), immunoglobulin M (IgM, P = 0.004), and a higher baseline neutrophil ratio (P <0.001). The baseline neutrophil ratio (r = 0.359, P = 0.001), IgM (r = −0.272, P = 0.025), and IL-6 (r = −0.259, P = 0.027) were significantly correlated with PIIRS. Combining principal component analysis and random forest results, neutrophil ratio, neutrophil count, IgM, IL-6, and D-dimer were useful predictors. The accuracy of random forest prediction was 75.00%, AUC, and sensitivity were 0.76 and 70%, respectively. Further survival analysis of the time from treatment to PIIRS revealed that the development of PIIRS was associated with IgM (more than 98 days of treatment) and neutrophil ratio/count.

**Conclusion:**

Baseline neutrophils ratio, neutrophil count, IgM, IL-6, and D-dimer may be clinically useful predictors of PIIRS in HIV-negative immunocompetent CM patients.

## Introduction

Cryptococcal meningitis (CM) is an important opportunistic infection worldwide. Each year, approximately 180,000 people die as a result of AIDS, accounting for 15% of AIDS-related deaths (1). HIV-related *Cryptococcus neoformans* infections have been relatively reduced due to highly active anti-retroviral therapy (HAART) (2). However, after the initiation of HAART, about 10 to 42% of HIV-CM patients developed new symptoms or deterioration of existing symptoms, and effective antifungal treatment is invalid, which are called cryptococcal associated immune reconstitution inflammatory syndrome (IRIS) (3). Similar immune responses can also occur in previously healthy individuals, which called postinfectious inflammatory response syndromes (PIIRS) (4). The clinical manifestations of PIIRS are similar to those of recurrence and persistent infection of CM (5). Therefore, the early identification and treatment of PIIRS in CM patients is essential.

In our previous studies (6, 7), we found that HIV-uninfected and nontransplanted male CM patients with PIIRS had increased rates of hearing loss, ventriculoperitoneal shunt (VPS), amphotericin B treatment, high cerebrospinal fluid (CSF) pressure, and cryptococcal count compared with patients without PIIRS (6). Further, we found that baseline hearing impairment and high CSF pressure (≥230 mmH_2_O) were the clinically useful predictors of PIIRS (7), but the study did not investigate the possibility of serum immune factors and inflammation-related cytological and biochemical features as predictors.

Therefore, we fill our previous research gap and firstly explore the serum immune-related predictors of PIIRS in HIV-negative immunocompetent CM patients in this study.

## Methods

### Study Design and Samples

This study is approved by the Medical Ethics Committee of the Third Affiliated Hospital of Sun Yat-sen University (approval no. [2021] 02-264-01). All study participants have provided written consent for research and publication.

**Figure 1:**
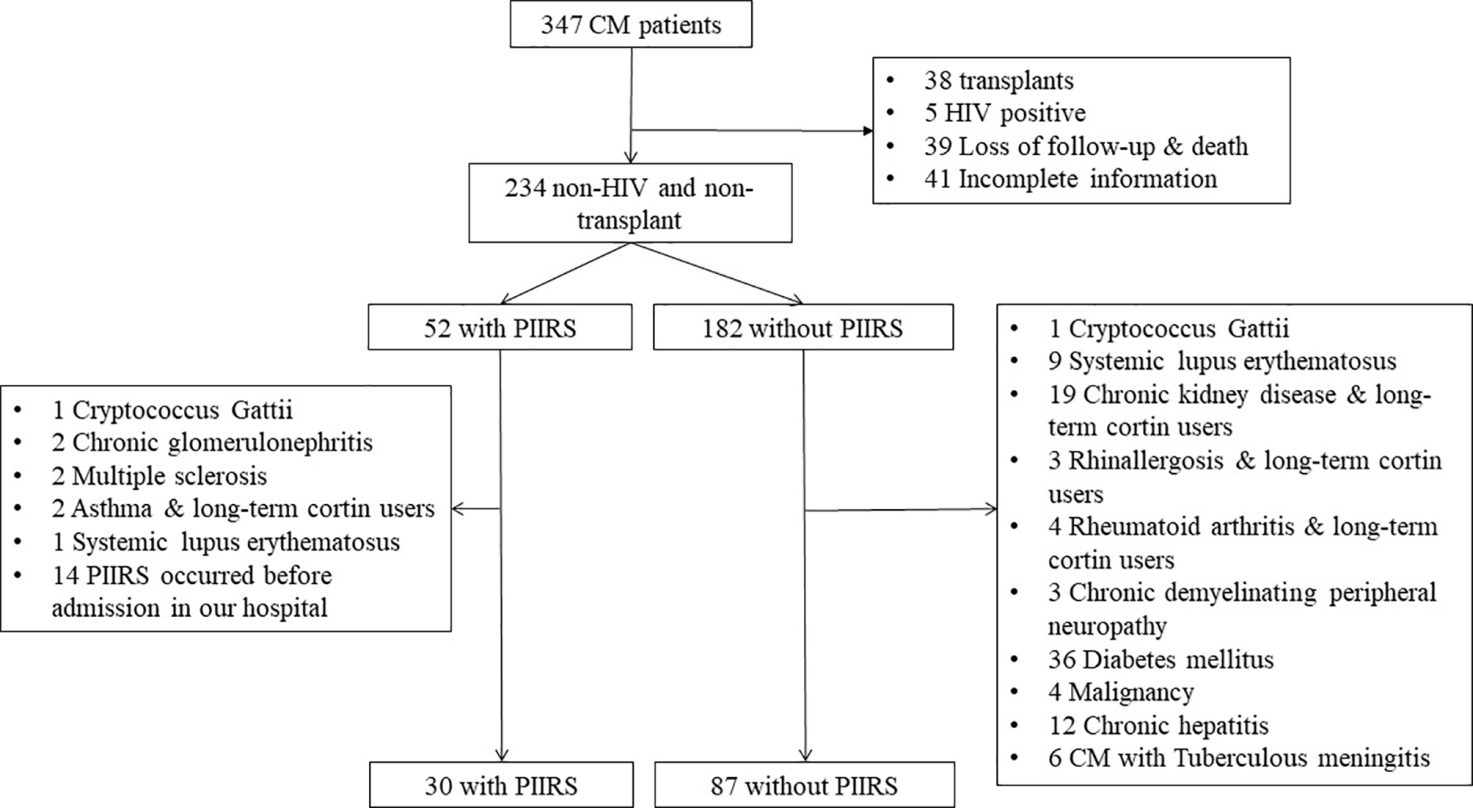
Flow chart of patient cohort recruitment. CM, cryptococcal meningitis; PIIRS, post-infectious inflammatory response syndrome; 19 Chronoc kidney diseases including 12 chronic glomerulonephritis, 5 nephrotic syndrome, 1 purpura nephritis, 1 diabetic nephropathy.

Data were derived from 347 Chinese Han CM patients enrolled between Jan 2011 and Dec 2020 at the Third Affiliated Hospital of Sun Yat-sen University, Guangzhou, China. Patients underwent physical and neurological examinations. A brain magnetic resonance imaging (MRI) scan and lumbar puncture were performed. The blood cytology, biochemical, and immunological tests were carried out. Finally, we recruited 117 patients with HIV-negative immunocompetent CM, including 30 with PIIRS and 87 without PIIRS. The inclusion and exclusion processes of diagnosis are shown in **Figure 1**. Patients with CM have been defined with clinical symptoms and a positive result of CSF culture or India ink stain for *C. neoformans* (8). PIIRS is defined in previously healthy people with CM, paradoxically, as the presence of clinical deterioration during effective antifungal treatment due to an exuberant immune response. Clinical manifestations include worsening or relapse of clinical symptoms and/or new MRI, and continued negative fungal cultures (1, 4). The demographic features, the course of disease, and the occurrence time of PIIRS were recorded. Additionally, serum immunological, cytological, and biochemical indexes were included in the analysis and used as variables to predict the onset of PIIRS.

### Treatment Strategies

Most patients with CM were treated with amphotericin B (AMB, 0.7 mg/kg/day) combined with 5-fluorocytosine (5-FC, 100 mg/kg/day, 4 times orally), followed by consolidation treatment with fluconazole (FLU, 400–600 mg/day) or voriconazole (VOR, 200 mg/day, 2 times) for 8 weeks, then FLU (200 mg/day) or VOR (200 mg/day) was maintained for at least 6 months (9).

### Laboratory Examination for Assessment

Baseline blood indexes at antifungal treatment initiation and PIIRS onset were recorded, namely, serum immunological indexes [interleukin-6 (IL-6), interleukin-10 (IL-10), tumor necrosis factor-α (TNF-α), complement 3 (C3), complement 4 (C4), 50% hemolytic unit of complement (CH50), immunoglobulin G (IgG), and immunoglobulin A (IgA)], serum cytological indexes, namely, neutrophil count (NETC) and neutrophil ratio (NETR), inflammatory indexes [procalcitonin (PCT), serum calcium (Ca), 25-hydroxyvitamin D (HVitD), and D-dimer (d-d)].

### Statistical Analysis and RF-Based Predictor Identification

For analyses, only the clinical data and laboratory examination data were included as candidate variables that might be predictors of PIIRS. We first analyzed the features of each variable in the baseline data and their differences between baseline and PIIRS onset. We used the two-sample or paired t-test for continuous variables that conform to normal distribution, the two-sample Wilcoxson rank-sum test or signed-rank test for continuous variables that do not follow a normal distribution, and the chi-square test for discrete variables. *P <*0.05 was considered statistically significant.

After that, to explore the influence of the variables on PIIRS, the data were standardized by Z-score. The correlation between variables and PIIRS was analyzed by Spearman correlation analysis. Then principal component analysis (PCA) analyzed the feature importance for PIIRS according to factor loads.

To identify the potential predictors for PIIRS, a random forest (RF) classifier was constructed and its performance was compared with seven other machine learning (ML)-based classifiers: gradient boosting decision tree (GBDT), CatBoost, AdaBoost, light gradient boosting machine (LGBM), extreme gradient boosting (XGB), decision tree (DT), and support vector machines (SVM). The datasets, including 117 patients recruited, were randomly divided into the training set and test set by 7:3. Following this, the trained classifier was used to predict the accuracy within the test set by calculating the false positive rate and false negative rate. The evaluation indexes include accuracy, sensitivity, specificity, F1 score, and the area under the ROC curve (AUC). The variables with a feature importance of more than 5% were potential predictors for PIIRS.

Finally, survival was estimated using the Kaplan–Meier method, and the comparison between study groups was performed using the log-rank test. The survival rate was calculated as the time from CM treatment to PIIRS onset. All tests were two-sided, and Log-rank P <0.05 was considered significant.

GraphPad prism (version 7.0) was used for parameter and nonparametric tests. Survival analyses were performed by R software. Spearman correlation analysis, PCA, and machine learning models were implemented by Python (version 3.7.3) with the scikit-learn package (version 0.23.2).

## Results

### Baseline Features of CM Patients With PIIRS and Without PIIRS

As shown in **Table 1**, CM patients with PIIRS (male:female = 22:8) were compared with CM patients without PIIRS (male:female = 67:22), with a median age of 39 years compared with 41 years. The median time from the first visit (equivalent to antifungal therapy initiation) to the onset of PIIRS was 57 (13–162) days. There were no differences in baseline levels of IL-10, TNF-α, C3, C4, CH50, IgG, IgA, NETC, PCT, Ca, HVitD, and d-d between the two groups. However, patients with PIIRS had lower IL-6 levels (P = 0.005), lower IgM levels (P = 0.003), and higher NETR (P <0.001) at baseline.

**Table 1:** Baseline demographic and clinical characteristics of HIV-negative cryptococcal meningitis patients with or without PIIRS.

Characteristic	HIV-negative CM with PIIRS	HIV-negative CM without PIIRS	*p*-value
Number	30	87	
Age, mean years (Std. Deviation)	39.37 (13.06)	42.08 (14.03)	0.177
Gender, n (%)			
male	22 (73.3)	66 (75.9)	0.809
female	8 (26.7)	21 (24.1)	0.809
Duration of disease*			
≤1 month	14 (46.7)	19 (21.8)	**0.028**
1–3 month	7 (23.3)	36 (41.4)	
≥3 month	9 (30.0)	32 (36.8)	
Time from diagnosis of CM to PIIRS, median days (range)	57 (13–162)	N/A	
**Serum immunological indexes**			
Interleukin-6 (IL-6), median pg/ml (range)**	6.52 (1.5–19.62)	7.44 (1.81–1415)	**0.006**
Interleukin-10 (IL-10), median pg/ml (range)	5(5–10.03)	5(5–17.2)	0.090
Tumor necrosis factor-α (TNF-α), median pg/ml (range)	11.08 (5.03–20.62)	10.9 (5.03–79.52)	0.667
Complement 3 (C3), median g/L (range)	1.31 (0.58–1.93)	1.37 (0.63–2.07)	0.394
Complement 4 (C4), median g/L (range)	0.3 (0.12–0.59)	0.35 (0.06–1.65)	0.796
Fifty percent hemolytic unit of complement (CH50), median U/ml (range)	57.5 (28–76)	57 (18–80)	0.776
Immunoglobulin G (IgG), median g/L (range)	11.12 (6.44–33.15)	12.97 (1.85–29.15)	0.158
Immunoglobulin M (IgM), median g/L (range)**	1.09 (0.21–3.44)	1.79 (0.39–4.15)	**0.004**
Immunoglobulin A (IgA), median g/L (range)	3.23 (0.69–6.23)	2.42 (0.55–6.57)	0.107
**Haemocytes & biochemistry**			
Neutrophil count (NETC), median ×10^9^/L (range)	7.40 (1.57–21.32)	6.1 (1.2–21.52)	0.093
Neutrophil ratio (NETR), median % (range)**	80.3 (48.3–92.1)	70.4 (10.4–94.3)	**0.0001**
Serum calcium (Ca), median mmol/L (range)	2.39 (1.87–2.64)	2.34 (1.34–2.87)	0.252
Procalcitonin (PCT), median ng/MI (range)	0.094 (0.043–0.472)	0.083 (0.024–2.53)	0.791
D-dimer (d-d), median μg/ml (range)	0.875 (0.22–16.6)	0.75 (0.14–11.67)	0.452
25-hydroxyvitamin D (HVitD), mean nM (SD)	69.74 (2.90)	64.48 (1.49)	0.156

Data was based on chi-square test, Two-sample Wilcoxon rank-sum test or Two-sample T-test; *p <0.05; **p <0.01. N/A: This variable (Time from diagnosis from CM to PIIRS) was not applicable for HIV-negative CM patients without PIIRS.

**Figure 2:**
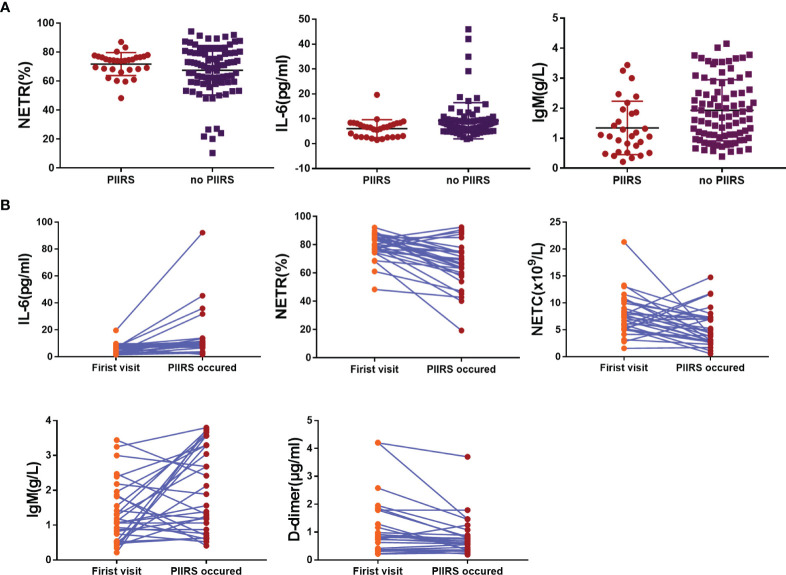
Trend of each variable with significant statistical differences in baseline data. **(A)** The scatter diagram showed the significant difference of NETR, IL-6, and IgM between the patients with PIIRS and without PIIRS. **(B)** The scatter diagram showed the significant difference of NETR, IL-6, NETC, IgM, and D-dimer in patients at baseline and PIIRS onset. Two-sample Wilcoxon rank-sum test was used for Data **(A)**; Wilcoxon signed-rank test was used for Data **(B)**; P <0.05 indicates statistical difference, and P <0.01 indicates significant statistical difference.

In the self-comparative analysis of CM patients with PIIRS (see **Table 2** and **Figure 2**), there were higher levels of IL-6 (P <0.001), IgM (P = 0.03), lower levels of NETR (P <0.001), NETC (P = 0.01), and d-d (P = 0.003) at the onset of PIIRS compared with their baseline levels.

**Table 2:** First visit (Baseline) vs PIIRS onset in HIV-negative patients with cryptococcal meningitis.

	First visit (Baseline)	PIIRS onset	*p*-value
**Serum immunological indexes**			
Interleukin-6 (IL-6), median pg/ml (range)**	6.52 (1.5–19.62)	9.8 (1.8–92.24)	<0.001
Interleukin-10 (IL-10), median pg/ml (range)	5 (5–7.35)	5 (5–23.6)	0.452
Tumor necrosis factor-α(TNF-α), median pg/ml (range)	11.08 (5.03–20.62)	12.68 (6.42–50.03)	0.191
Complement 3 (C3), median g/L (range)	1.31 (0.58–1.93)	1.35 (0.64–1.96)	0.551
Complement 4 (C4), median g/L (range)	0.3 (0.12–0.59)	0.31 (0.11–0.55)	0.821
Fifty percent hemolytic unit of complement (CH50), median U/ml (range)	57.5 (28–76)	59 (25–77)	0.578
Immunoglobulin G(IgG), median g/L (range)	11.12 (6.44–33.15)	11.52 (6.09–30.84)	0.478
Immunoglobulin M(IgM), median g/L (range)*	1.09 (0.21–3.44)	1.73 (0.41–3.8)	0.03
Immunoglobulin A(IgA), median g/L (range)	3.23 (0.69–6.23)	3.33 (0.32–6.3)	0.918
**Hemocytes & biochemistry**			
Neutrophil count (NETC), median ×10^9^/L (range)*	7.40 (1.57–21.32)	4.39 (0.57–17.82)	0.010
Neutrophil ratio (NETR), median % (range)**	80.3 (48.3–92.1)	66.6 (19.2–92.4)	<0.001
Serum calcium (Ca), median mmol/L (range)	2.39 (1.87–2.64)	2.38 (2.1–2.8)	0.360
Procalcitonin (PCT), median ng/MI (range)	0.094 (0.043–0.472)	0.081 (0.024–0.895)	0.262
D-dimer (d-d), median μg/ml (range)**	0.875 (0.22–16.66)	0.67 (0.19–13.81)	0.003
25-hydroxyvitamin D (HVitD), mean nM (SD)	69.74 (2.90)	70.58 (3.44)	0.426

Data was based on Wilcoxon signed-rank test or Paired T-test; *p <0.05; **p <0.01.

### Factors Associated With PIIRS of CM Patients at Baseline

**Figure 3:**
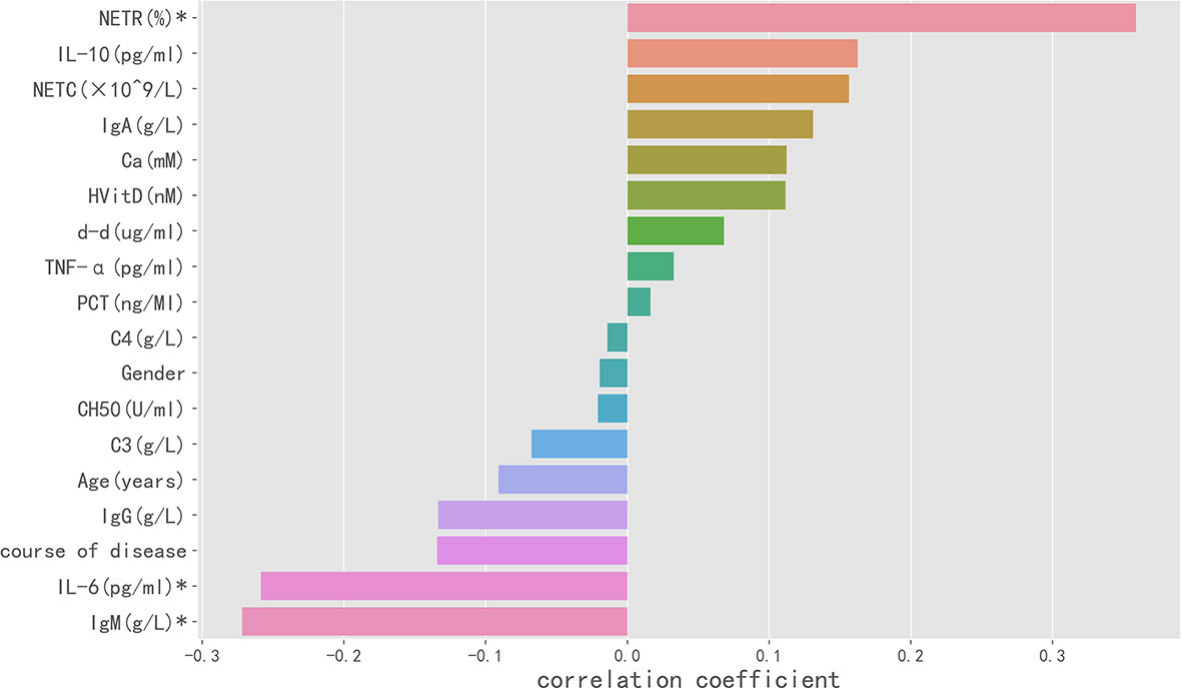
Spearman correlation analysis indicated the variables with strong correlation with PIIRS. The horizontal histogram shows the correlation between the study variables and PIIRS, the longer the column, the higher the correlation. Negative values of the abscissa indicate negative correlation, whereas positive values indicate positive correlation. * marked variables with significant statistical differences (p <0.05).

**Figure 4:**
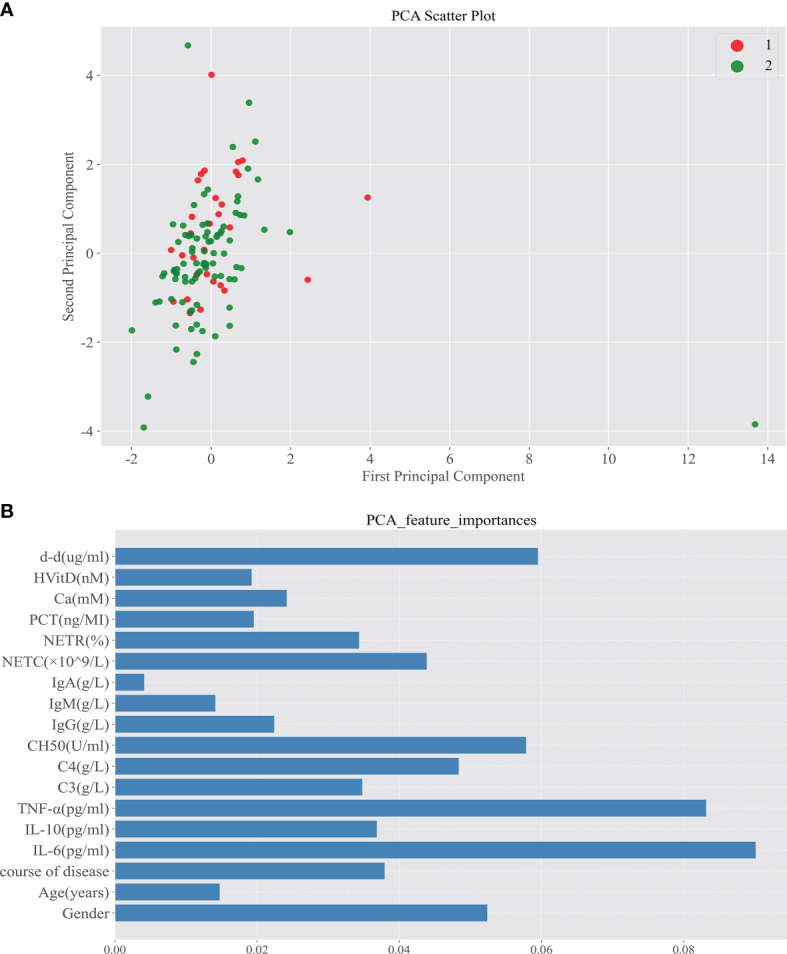
Principal component analysis screened out the important characteristic variables associated with the development of PIIRS. **(A)** After reducing the sample to two-dimensional through PCA, observe the projection distribution of the sample in the first two principal components of PCA; Red dot 1 represents patients with PIIRS and green dot 2 represents patients without PIIRS; **(B)** We analyze the feature importance of the first two dimensions The feature importance is shown in the panel **(B)**, the longer the column, the higher the importance.

In Spearman correlation analysis, baseline NETR (r = 0.359, P = 0.001), IgM (r = −0.272, P = 0.025), and IL-6 (r = −0.259, P = 0.027) were significantly correlated with PIIRS (see **Table 3** and **Figure 3**). In principal component analysis, the importance ranking of the variable features associated with PIIRS was IL-6, TNF-α, d-d, NETC, CH50, and gender. After data dimensionality reduction, the projection distribution of PIIRS as the principal component in PCA is shown in **Figure 4A**, and the ranking of feature importance is shown in **Figure 4B**.

**Table 3:** The specific situation of Spearman correlation analysis on PIIRS onset.

Variable	Correlation coefficient (r)	P-value	P_adj_ value	The order of *p*-value
NETR (%)	0.358897028	6.11939E−05	0.001101491	1
IgM (g/L)	−0.27183884	0.00278393	0.025055371	2
IL-6 (pg/ml)	−0.258594877	0.00451661	0.027099659	3
NETC (×10^9^/L)	0.156349505	0.089502415	0.322208694	4
IL-10 (pg/ml)	0.162516882	0.07741324	0.322208694	5
IgA (g/L)	0.130989234	0.155617251	0.350138814	6
IgG (g/L)	−0.133522132	0.147709135	0.350138814	7
course of disease	−0.13430705	0.145321093	0.350138814	8
HVitD (nM)	0.111558676	0.227089365	0.408760858	9
Ca (mM)	0.112427043	0.223470104	0.408760858	10
Age (years)	−0.091032479	0.324815011	0.531515472	11
C3 (g/L)	−0.06761156	0.465019525	0.643873189	12
d-d (ug/ml)	0.068179846	0.461267296	0.643873189	13
C4 (g/L)	−0.0140882	0.879132139	0.879132139	14
PCT (ng/MI)	0.016339926	0.859997101	0.879132139	15
Gender	−0.019475655	0.833486816	0.879132139	16
CH50 (U/ml)	−0.020855775	0.821876047	0.879132139	17
TNF-α (pg/ml)	0.032676477	0.72424589	0.879132139	18

P-values were adjusted for multiple testing with false discovery rate (FDR) step-up method. NETR, Neutrophil ratio; IgM, Immunoglobulin M; IL-6, Interleukin-6; NETC, Neutrophil count; IL-10, Interleukin-10; IgA, Immunoglobulin A; IgG, Immunoglobulin G; HvitD, 25-hydroxyvitamin D; Ca, Serum calcium; C3, Complement 3; d-d, D-dimer; C4, Complement 4; PCT, Procalcitonin; CH50, Fifty percent hemolytic unit of complement; TNF-α, Tumor necrosis factor-α.

### Prediction of PIIRS by Random Forests

**Figure 5:**
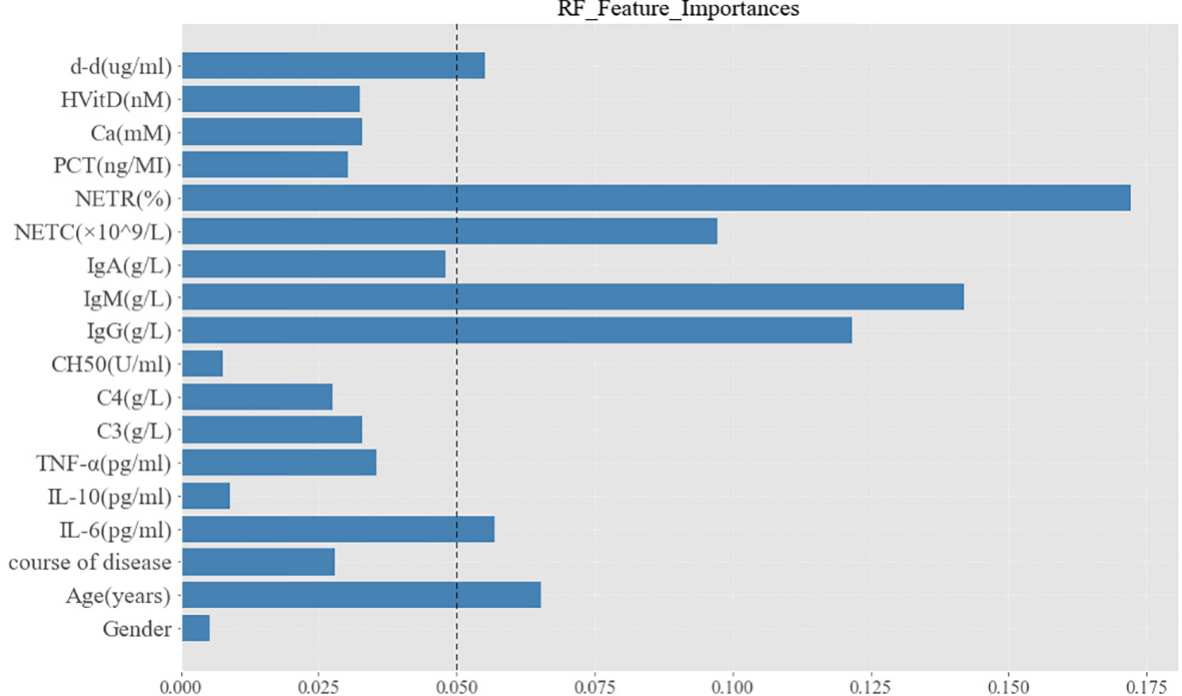
Characteristic importance of random forest for PIIRS prediction. The variables with feature importance more than 0.05 were regarded as potential predictors to PIIRS.

A variety of ML models were used to predict the onset of PIIRS. The prediction performance and ROC curve are shown in **Table S1** and **Figure S1**. The accuracy of RF prediction was 75.00%, the average F1 value was 0.7125; the AUC and sensitivity were 0.76 and 70%, respectively. Among the 18 candidate predictors, the feature importance ranking was as follows: NETR, IgM, IgG, NETC, age, d-d, and IL-6. The functional importance of RF is shown in **Figure 5**. Based on the above Spearman correlation analysis and PCA results, NETR, NETC, IgM, IL-6, and d-d were useful predictors of PIIRS.

### The Time From the Antifungal Treatment to PIIRS Onset

**Figure 6:**
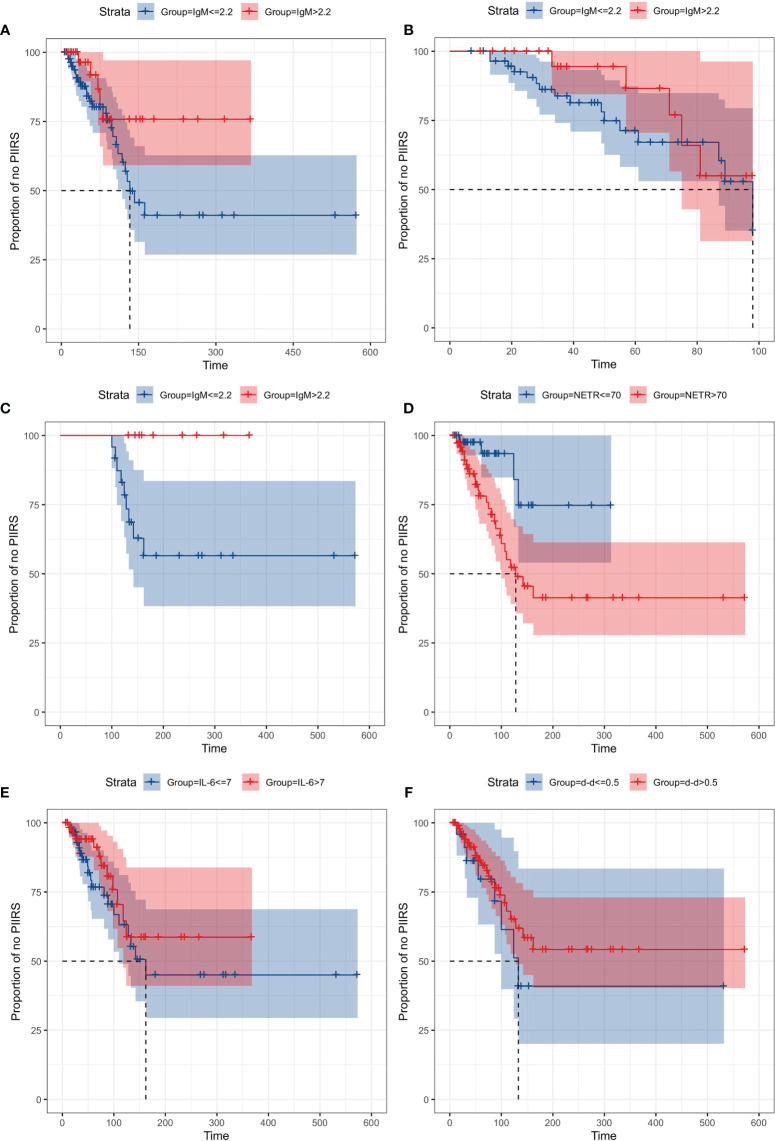
Kaplan–Meier curves of time from treatment to PIIRS, grouped by IgM, NETR, IL-6, and d-d. Cases of PIIRS were observed at the jump points of the curves, with maximum 600 days of follow-up. Each variable was grouped by the maximum value of the reference range. **(A)** IgM (≤2.2 g/L) vs IgM (>2.2 g/L) from treatment to PIIRS; **(B)** IgM (≤2.2 g/L) vs IgM (>2.2 g/L) from treatment to PIIRS when the follow-up time was less than 98 days; **(C)** IgM (≤2.2 g/L) vs IgM (>2.2 g/L) from treatment to PIIRS when the follow-up time was more than 98 days; **(D)** NETR (≤70%) vs NETR (>70%) from treatment to PIIRS; **(E)** IL-6 (≤7 pg/ml) vs IL-6 (>7 pg/ml) from treatment to PIIRS; **(F)** d-d (>0.5 μg/ml) vs d-d (≤0.5 μg/ml) from treatment to PIIRS.

The Kaplan–Meier curves for variables (IgM, NETR, IL-6, and d-d) from antifungal treatment to PIIRS are shown in **Figure 6**. Due to the high correlation between NETC and NETR, only NETR was selected for survival analysis, and each variable was grouped by the maximum value of the reference range (10–13). The survival curve of the IgM group had an intersection at about 98 days, and 98 days was set as the segmentation point for survival analysis. Interestingly, when the follow-up time was more than 98 days, patients with IgM ≤2.2 g/L were more likely to develop PIIRS than patients with IgM >2.2 g/L(log rank test, P = 0.035). Additionally, patients with higher NETR (>70%) may develop PIIRS 14.5 days faster than patients with lower NETR (≤70%) (log rank test, P = 0.01).

## Discussion

Studies on HIV-positive CM-IRIS showed that IRIS could be predicted by immune and inflammatory biomarkers (such as IL-6, TNF-α, IFN-γ, CD4 cell count, etc. before ART) (14–16). However, these findings may not apply to HIV-negative immunocompetent CM-PIIRS. To the best of our knowledge, this is the first study of serum immunological indexes to predict the onset of PIIRS in HIV-negative immunocompetent CM. We found that baseline NETR was significantly positively correlated with PIIRS, and baseline IL-6 and IgM were significantly negatively correlated with PIIRS onset. Principal component analysis and RF prediction models showed that NETR, NETC, IgM, IL-6, and d-d were useful predictors of PIIRS. Further survival analysis of the days from baseline to PIIRS onset suggested that the development of PIIRS was associated with serum IgM (more than 98 days of treatment). PIIRS occur faster in CM patients with high NETR (>70%) than in those with low NETR (≤70%).

Serum IL-6 increased significantly when HIV-related CM-IRIS occurred (17). Our study also found that IL-6 increased significantly at the PIIRS onset, which indicated a robust Th1 immune response at this time (4). For healthy individuals, the potential damage of PIIRS was usually caused by type 1 helper T cell (Th1) activation, which was manifested as related soluble markers (such as sCD27, IFN-γ, and IL-6) expressed *in situ* (4). In contrast, the low baseline IL-6 level in patients with PIIRS may indicate delayed Th1 immune response and high fungal burden (18). In this study, we also found that there was a negative correlation between baseline high levels of IL-6 and the onset of PIIRS, and low baseline IL-6 was a risk factor for the onset of PIIRS. However, survival analysis found no differences in IL-6 of different quantitative standards (>7 pg/ml vs 0–7 pg/ml) during baseline to PIIRS. In addition, we did not find a significant reduction in baseline TNF-α at PIIRS onset. Low levels of TNF-α represents an inefficient clearance of fungi and increased risk of IRIS for the HIV-related CM (14). Although TNF-α has not been identified as a predictor by RF, we considered that serum TNF-α may have important clinical significance for PIIRS onset. According to the results of principal component analysis, TNF-α was an important variable features associated with the PIIRS second only to IL-6. TNF-α classified as a non-predictor in the RF results may be due to the limitation of sample size. Therefore, more studies are needed to confirm the effect and the role of serum TNF-α in CM-PIIRS.

Neutrophils have received little attention in previous studies, and their role in cryptococcosis is usually controversial (19). A high neutrophil count and neutrophil/lymphocyte ratio at baseline suggested a poor prognosis of HIV-positive CM (20, 21). Animal studies have shown that blood-derived monocytes and neutrophils help *C. neoformans* cross the microvascular endothelial barrier through a phagocyte dependent pathway (19). Therefore, neutrophils in the peripheral blood after fungal infection may play an important role in the pathophysiology of CM-PIIRS. In this study, we found that baseline peripheral blood NETR in CM-PIIRS patients were significantly higher than those in CM patients without PIIRS. Additionally, the PIIRS event had occurred earlier in HIV-negative immunocompetent CM patients with a baseline peripheral blood NETR higher than 70%, which indicated that patients with high baseline NETR may have high CNS fungal transport through a phagocyte-dependent pathway associated with neutrophils and phagocyte in the early stage of infection. This situation may reflect the role of neutrophils in the early stage of cryptococcal infection before PIIRS onset.

Generally, the host immune response of HIV-negative CM is mostly mediated by T cells, and cell-mediated immunity seems critical for cryptococcal clearance (22). Nevertheless, several studies indicate the importance of humoral immunity for protection from cryptococcal infection (23). Various studies demonstrated the ubiquitous presence of anti-cryptococcal IgM and IgG antibodies in human serum directed against cryptococcal antigens (24, 25), regardless of previous history of cryptococcal disease or HIV infection. Anti-cryptococcal IgM antibodies ubiquitously present in the serum are believed to mainly target polysaccharides of the cell wall (26). These IgM antibodies play an important role in the defense against cryptococcal infection. In the early stages of CM, we found lower baseline serum IgM levels in patients with PIIRS. In terms of mechanism, the onset of PIIRS is associated with the continuous expression of cryptococcal antigen in local tissues (21). Therefore, we considered that IgM mediated complement dependent cytotoxicity and phagocytosis enhancement, which could eliminate *Cryptococcus* and neutralize cryptococcal antigen, to prevent the development of PIIRS. CM patients with IgM (>2.2 g/L) were less likely to have PIIRS development after 98 days, so baseline high IgM indicated a low risk of PIIRS development during the treatment.

The increase of serum d-d was observed in HIV-positive IRIS events. High serum d-d after ART suggested an increased risk of IRIS (27), but its significant increase was more common in non-central nervous system IRIS events (28). In our study, baseline serum d-d could be used as a useful predictor of PIIRS, suggesting that patients with higher serum d-d at baseline may be more prone to PIIRS. We also found that the decrease of serum d-d in PIIRS events compared with baseline was contrary to the d-d trend of HIV-positive IRIS. The immunological mechanism of d-d for PIIRS has not been clarified, and the evidence of clinical significance is not sufficient, which needs to be further studied.

There are several limitations to this study. It was a retrospective study from a single center and a single ethnic population. Some variables were incomplete or not available. For example, baseline serum cryptococcal antigen (CrAg) titer, Th1 activation related cytokines, such as IFN-γ, interleukin-1, chemokine ligand 2, C-X-C motif chemokine ligand 10 (14, 15), and Th17 activation related cytokines, such as interleukin-17 (29) were not available in our study. Besides, the numbers of patients were relatively small. However, to the best of our knowledge, this is the first study to investigate serum immune factors for predicting the development of PIIRS. Our hospital is the major research unit focused on this condition in China. In the future, we will collect more patients for further investigation.

## Conclusion

Baseline neutrophil ratio, neutrophil count, IgM, IL-6, and D-dimer may be clinically useful predictors of PIIRS in HIV-negative immunocompetent CM patients. Patients with high NETR (>70%) and IgM (0–2.2 g/L) (more than 98 days of treatment) were more likely to develop PIIRS during antifungal therapy. We should pay attention to those CM patients with risk factors for the development of PIIRS.

## Data Availability Statement

The raw data supporting the conclusions of this article will be made available by the authors, without undue reservation.

## Ethics Statement

The studies involving human participants were reviewed and approved by the Medical Ethics Committee of the Third Affiliated 75 Hospital of Sun Yat-sen University. The patients/participants provided their written informed consent to participate in this study.

## Author Contributions

Conception and design of this study: YW, YJ, and FP. Data collection and organization: YW, LS, JiL, ML, XX. Data analysis: HW, YW, LS, and XS. Manuscript drafting: YW, HW, LS, YJ, and FP. Figure and table preparation: YW, HW, XS, XX, and ML. Manuscript reviewing: YW, YJ, and FP. Methods & Data rechecking: HW and YW. Quality control of case data: LS, JiL, XX, YW, YJ, and FP. All authors listed have made a substantial, direct, and intellectual contribution to the work and approved it for publication.

## Funding

This study is supported by the general program of the China Postdoctoral Science Foundation (No. 2019M660230).

## Conflict of Interest

The authors declare that the research was conducted in the absence of any commercial or financial relationships that could be construed as a potential conflict of interest.

## Publisher’s Note

All claims expressed in this article are solely those of the authors and do not necessarily represent those of their affiliated organizations, or those of the publisher, the editors and the reviewers. Any product that may be evaluated in this article, or claim that may be made by its manufacturer, is not guaranteed or endorsed by the publisher.
