# Supplementary material for: Immunological Predictors of Post Infectious Inflammatory Response Syndrome in HIV-Negative Immunocompetent Cryptococcal Meningitis
Source: Front Immunol. 2022 May 24;13:895456. doi: 10.3389/fimmu.2022.895456 (PMC9171325; doi:10.3389/fimmu.2022.895456)
Supplement: Supplementary file 1 [file DataSheet_1.docx]

**Supplementary Table 1 Prediction performances of different models**

| **Model** | **Accuracy (%)** | **Specificity (%)** | **Sensitivity (%)** | **Average F1** | **AUC** |
| --- | --- | --- | --- | --- | --- |
| GBDT | 66.67 | 76.92 | 40.00 | 0.5846 | 0.67 |
| CatBoost | 75.00 | 96.15 | 20.00 | 0.5776 | 0.82 |
| DT | 63.89 | 76.92 | 30.00 | 0.5353 | 0.54 |
| SVM | 69.44 | 88.46 | 20.00 | 0.5368 | 0.70 |
| AdaBoost | 75.00 | 84.62 | 50.00 | 0.6783 | 0.63 |
| LGBM | 72.22 | 76.92 | 60.00 | 0.6727 | 0.71 |
| XGB | 77.78 | 88.46 | 50.00 | 0.7037 | 0.74 |
| RF | 75.00 | 76.92 | 70.00 | 0.7125 | 0.76 |

**Note: RF: random forest; GBDT: gradient boosting decision tree; CatBoost: gradient boosting with categorical features support; LGBM: light gradient boosting machine; DT: decision tree; SVM: support vector machine; XGB: extreme gradient boosting.**


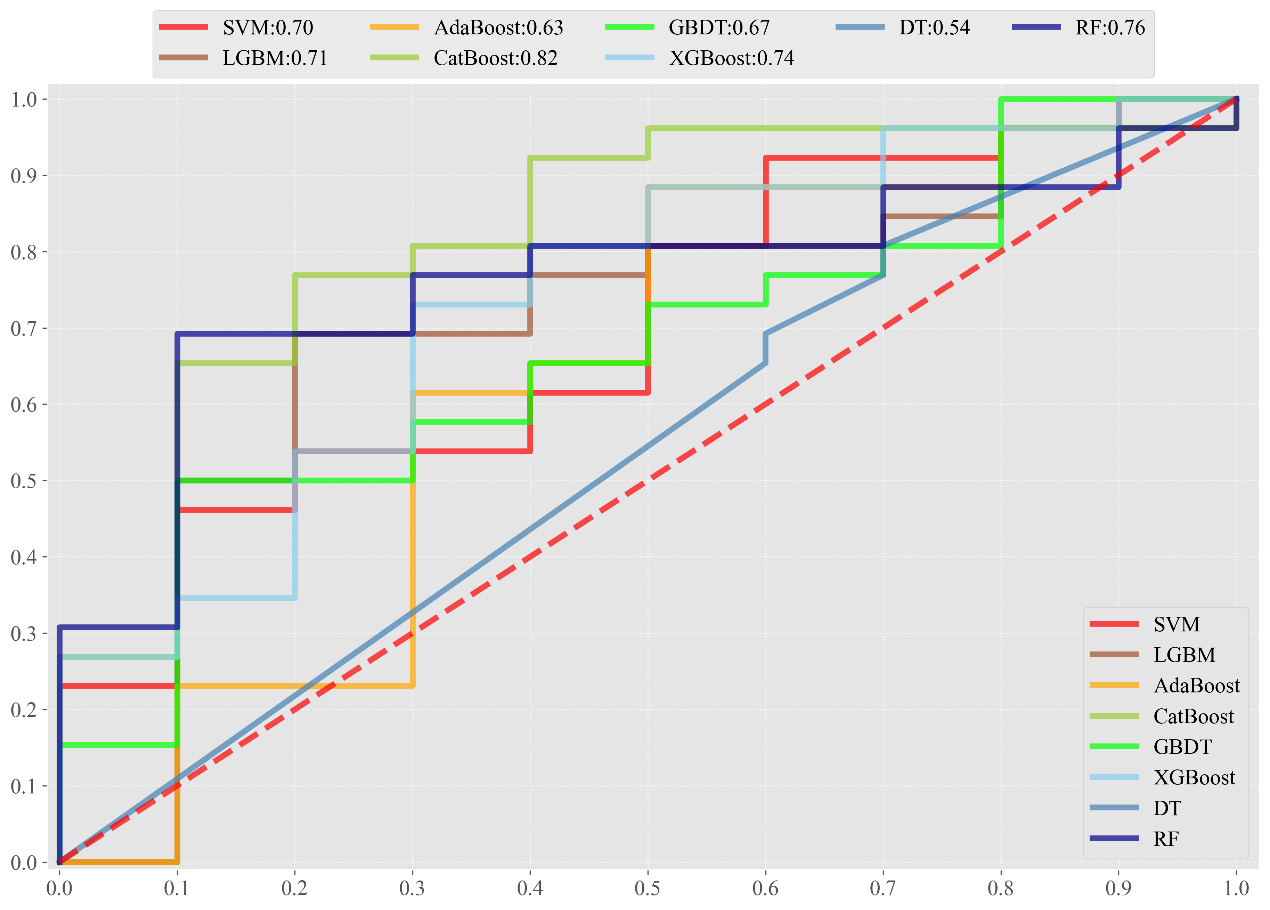


**Supplementary Figure 1. ROC curves of different prediction models for PIIRS prediction**
